# A qualitative exploration of stressors in anaesthesia training in the UK and mechanisms to improve resident wellbeing

**DOI:** 10.1111/anae.16575

**Published:** 2025-02-25

**Authors:** Thomas Gale, Sophie Winter, Harriet Daykin, John Tredinick‐Rowe, Lyndsey Withers, Marie Bryce

**Affiliations:** ^1^ Collaboration for the Advancement of Medical Education Research, Peninsula Medical School University of Plymouth Plymouth UK; ^2^ Department of Anaesthesia University Hospitals Plymouth NHS Trust Plymouth UK; ^3^ Department of Anaesthesia Royal Devon Healthcare NHS Foundation Trust Exeter UK; ^4^ Patient representative University of Plymouth Plymouth UK

**Keywords:** anaesthesia, stress, training, wellbeing

## Abstract

**Introduction:**

High levels of stress and burnout have been identified among resident anaesthetists in UK training programmes. Factors involving clinical roles, workplace culture and training are known stressors, but in‐depth research investigating how to improve wellbeing is limited.

**Methods:**

We used a qualitative design in two phases with participants from across the UK. Phase 1 involved semi‐structured interviews of resident anaesthetists in the 2nd–5th years of training, and educational stakeholders. Phase 2 involved additional participants in two focus groups, one each for residents and stakeholders. Interviews and focus groups were conducted online, audio‐recorded and transcribed for thematic analysis using a framework approach.

**Results:**

We interviewed 52 participants in phase 1, comprising resident anaesthetists from England, Wales and Scotland and key educational stakeholders. A further 11 resident anaesthetists and stakeholders participated in the phase 2 focus groups. We identified four overarching themes contributing to stress: clinical work; non‐clinical work; structure of training; and workplace culture. We also identified supportive features at individual, local, regional and national levels. Stress and burnout were commonplace, particularly during demanding periods of training. Balancing non‐clinical commitments alongside busy workloads was difficult. Clinically, intensive care medicine and obstetrics generated the most stress. Frequent rotations and long commutes increased stress, impacting on working and family relationships. Curriculum changes, examinations and competition for higher training posts caused stress and poor morale. Proposed mechanisms to improve wellbeing include: peer‐to‐peer support; request‐based rotas; adoption of ‘lead employers’; decreasing rotation frequency and commuting distances; access to less than full‐time working and professional support; and adapting the structure of training to improve the stability of the resident anaesthetist workforce.

**Discussion:**

Attention to the factors identified as contributing to stress could improve resident anaesthetists' wellbeing through changes to policy and practice at local, regional and national levels, for which we make research‐informed recommendations.

## Introduction

Anaesthesia is a high‐pressure specialty, involving acute and challenging clinical situations. In UK anaesthetic training, close clinical supervision is required initially but this is often stepped down at a relatively early stage. The experience of stress in resident anaesthetists can affect their wellbeing and clinical performance. A recent scoping review of the literature found that external factors, such as training, clinical roles, progression, work patterns, access to support and workplace culture, were identified more frequently as affecting the wellbeing and stress levels of resident anaesthetists than individual factors like pre‐existing health status [1]. Surveys have identified excessive work‐related stress in residents, connected to the clinical and non‐clinical burden of training commitments [2, 3, 4], bringing a high risk of burnout [5]. The prevalence of stress has been identified as being between 19% and 37%, whilst burnout risk varies from 25% to 85% [2, 3]. The highest levels of risk have been identified consistently in the second, fourth and fifth years of training by the General Medical Council's National Training Survey [6, 7]. These are important transition points in anaesthesia training, with resident anaesthetists assuming additional clinical responsibilities with less direct supervision and confronting the hurdles of professional examinations.

Fatigue is common, and may impact physical health, psychological wellbeing and personal relationships [4]. Other stressors include the demands of non‐clinical workloads; exhaustion from multiple commitments; the burden of training; and changes in societal views of doctors [8]. Perhaps inevitably, the COVID‐19 pandemic also impacted resident doctors due to service delivery demands and disruptions to rotations, examinations and career progression [9]. The wellbeing of anaesthetic residents is of paramount importance to promote the retention of doctors within the specialty, and to maintain anaesthesia as an attractive career choice.

To date, the extent of in‐depth qualitative research to understand the experiences of resident anaesthetists has been limited. Most previous studies have used survey‐based research to quantify the prevalence of stress and risk of burnout, with less attention given to understanding these issues, or to improving support mechanisms in training programmes [1].

Our study, conducted from 2022 to 2024 against a backdrop of industrial action and widespread dissatisfaction among resident doctors, aimed to understand the stressors faced at key transition points in anaesthesia training and the effects of these on resident anaesthetists, and to identify support mechanisms and develop suggestions for improvements.

## Methods

This study used a qualitative design with two phases, comprising semi‐structured interviews (phase 1) and focus groups (phase 2). Ethical approval was granted by the University of Plymouth Faculty of Health Research Ethics and Integrity Committee.

A call for participants was circulated by the Royal College of Anaesthetists (RCoA) and the Association of Anaesthetists in November 2022. Potential participants were asked to provide demographic and professional characteristic information, which enabled us to sample participants purposively to promote diversity in terms of sex, ethnicity, place of primary medical qualification and geographic region (Table 1). All participants in phases 1 and 2 completed a Joint Information Systems Committee Online Survey form to give informed consent to joining the study [10].

**Table 1:** Summary of phase 1 participant characteristics.

	Sex		Ethnicity	
	Female	Male	Non‐white	White
Year 2	4	6	4	6
Year 3	6	4	3	7
Year 4	5	5	3	7
Year 5	6	4	4	6
Other	2	2	0	4
Stakeholders	3	5	1	7

For the phase 1 interviews, we recruited resident anaesthetists in years 2–5 of training: years 2, 4 and 5 have been identified as having a higher burnout risk [6, 7]; and year 3 has become a key transition point in the newer 2021 curriculum. Our sampling strategy was to include 10 resident anaesthetists from each of the four training year groups for interview, plus 10 educational stakeholders. We anticipated, based on our collective previous experience of qualitative research, that this sample would deliver sufficiently rich data to address our research questions and be achievable within the study timeframe. Within these proposed samples, we intended to seek balance in sex; training region within the UK; ethnicity; nationality (UK or non‐UK); place of primary medical qualification (UK or non‐UK); disability status; and whether in full or less than full‐time training. We received over 100 responses to the initial call for participants from resident anaesthetists, allowing us to select a sample according to these requirements.

During the participant recruitment phase, we were contacted by several people who had recently left their anaesthetics training programme before reaching consultant status. After discussion among the research team, we decided to include a small sample of these doctors as participants, as their experiences and reasons for leaving training potentially offered additional important insights relevant to our research questions.

The educational stakeholders whom we recruited included educational and clinical supervisors; RCoA college tutors; Training Programme Directors; and heads of postgraduate schools of anaesthesia. Educational and clinical supervisors are responsible for individual supervision and management of resident anaesthetists training needs; college tutors act as educational leads for anaesthesia at a departmental or hospital level; whilst training programme directors and heads of postgraduate schools hold responsibility at a regional level.

For phase 2, a second call for participants was circulated in September 2023, to recruit residents and stakeholders not involved with phase 1 to take part in focus groups.

Interviews were conducted remotely on Zoom (version 6.2.10, Zoom Communications Inc., San Jose, CA, USA) between December 2022 and April 2023 by three members of the research team (TG, SW and HD) using topic guides developed from the research questions (online Supporting Information Appendices S1 and S2). Interviews lasted 30–60 min each and were audio‐recorded and transcribed for analysis.

Two focus groups were held in November 2023 remotely and lasted 90 min each. Discussions were structured around themes emerging from analysis of the interview data. The resident focus group was facilitated by the study's resident co‐investigators only (SW and HD) as the research team felt their status as peers to the participants would engender greater openness, recognising the potential for the presence of a senior anaesthetist (TG) to inhibit participants from discussing some issues freely. The stakeholder focus group was facilitated by the researcher with the most experience as a trainer/educator (TG). The patient representative (LW) also participated in the focus groups. The focus groups were audio‐recorded and transcribed for analysis.

Data were coded by four members of the research team (SW, HD, TG and JT‐R) using Nvivo (version 14, Lumivero LLC, Burlington, MA, USA) and analysed thematically to elicit stressors faced by resident anaesthetists. We used framework analysis to structure the coding of transcripts [11]. This involves the development of a coding framework that is then used to code or categorise the data. We undertook an initial calibration exercise, in which those coding the data reviewed and coded the same five transcripts and developed an initial coding framework that was then applied to the remaining data, with codes added, merged or removed through discussion as the analysis progressed. Through discussion among the whole research team, codes were then drawn into categories and wider themes identified. Subsequently, the findings from this initial analysis of the interview data were reviewed, sense‐checked and refined in the focus groups. These focus group discussions also provided additional rich, confirmatory data, plus some novel insights and were coded using the same framework as the interview data. Our understanding of the themes was refined further following the focus groups, which expanded on the themes raised in the interview rather than refuting any earlier findings. Throughout the analysis phase of the study, the whole research team discussed the findings regularly, the development of broader themes from the coded data and how those themes related to each other.

The research team was composed of a mix of senior (TG) and early career (SW and HD) academic anaesthetists, social scientists with experience in qualitative health workforce research (JT‐R and MB) and a patient representative (LW). This mix of perspectives enriched the analysis, bringing in‐depth knowledge of anaesthetics training and work, but also included external perspectives. The anaesthetists in the team, all of whom have participated in UK anaesthetic training as learners and, in the case of TG, as an educator and curriculum developer, were careful to consider their own biases and the influence of their own experiences on their interpretations of the data. In addition, the other members of the team questioned assumptions and suggested alternative interpretations. In particular, the patient representative (LW), who contributed to the study design, development of topic guides and interpretation of the data, brought a different perspective to the study and ensured that we considered the wider implications of the issues identified.

## Results

We provide quotations from participants, grouped around the major themes identified in our analysis. Perspectives on anaesthetic careers are explored, along with the effects of stress on resident anaesthetists. Causes of stress have been grouped into non‐clinical; clinical; structural; and cultural themes. Supportive factors have been categorised from individual to national levels. Illustrative quotes covering all the major themes and subthemes emerging from our research have been collated in online Supporting Information Appendix S3.

Of the 44 resident anaesthetists recruited for interview, 37 worked in England (from 10 different training regions), five in Wales and two in Scotland. Residents were aged between 28 y and 44 y and 10 were in less than full‐time training. Eight reported having one or more long‐term physical or mental health conditions. Nine residents were born outside the UK, with six having completed their primary medical qualification outside the UK. All of the year 5 residents had passed the second part of the fellowship examination of the RCoA.

Stakeholder interviewees were aged between 41 y and 57 y, and all worked in England across five training areas. Their roles included Training Programme Directors; heads of postgraduate schools of anaesthesia; regional advisors; and educational supervisors. Their experience supporting resident anaesthetists ranged from 5 years to 22 years.

For the phase 2 resident anaesthetist focus group, we recruited five participants spread across training stages in training years 2, 3, 4 and 5; three male and two female. Our stakeholder focus group comprised six participants, two male and four female, with between 5 years and 16 years experience of supporting resident anaesthetists.

### Effects and sources of stress

Resident anaesthetists described effects of stress including fatigue; low mood; depression; sleep disturbances; and burnout. Tragically, more than one trainee had been directly affected by the suicide of an anaesthetic colleague. It was felt that better signposting was needed to support trainees in an acute crisis and for colleagues who may also be affected. Participants described the negative effects that training can have on their partners, children and friends. Additionally, compassion fatigue and reduced motivation, highlight how stress can also affect clinical performance.

*“I was constantly exhausted. You can't think straight a lot of the time, talk or enjoy doing the work you're doing. You hate going to work, feeling low in mood and kind of stressed, and like small things just stress you out and I get irritable very quickly.”* P030, year 4 resident anaesthetist, female

We identified four themes relating to sources of stress, which are described below.

### Non‐clinical commitments

Professional examinations (specifically the fellowship examination of the RCoA) were often found to be stressful due to the wide curriculum covered; perceived lack of relevance of some content; the amount of revision required; and balancing examination preparation with clinical work and personal life.

*“The FRCA* [fellowship examination of the RCoA] *was definitely harder just because of the breadth of knowledge expected and in the level of detail that's required”* P021, year 4 resident, male

*“It's a lot of esoteric knowledge which doesn't actually help in your day to day*” Focus group 1, year 4 resident anaesthetist, male

The time commitment affected participants' work–life balance and ability to engage in activities benefiting wellbeing, such as socialising and exercise. These issues led many resident anaesthetists to resent the current approach to the fellowship examination of the RCoA, but some stakeholders felt that this was partly due to poor communication or recognition of the importance of certain topic areas and an outdated understanding of what will be examined.

*“The basis of this is a test of do you understand the devices, equipment, the drugs, the physiology around you, because understanding those prevents some of the awful mistakes I've seen in other parts of medicine that are based around a fundamental misunderstanding of the kit or the drugs people are using.”* Focus group 2, Training Programme Director, male

Examinations were particularly challenging for resident anaesthetists who had care commitments and those with neurodiversity. The format of the structured oral examination (a concise form of viva voce) was specifically identified as a stressor due to a lack of experience with this approach from undergraduate medical studies. Its proposed removal by the RCoA has been received positively. Failing the examination can significantly impact residents' perception of themselves, precipitating a ‘negative spiral’ or a lack of progression beyond core training.

Completing logbooks and online portfolios was deemed an extra burden. The competitive portfolio assessment for entry to year 4 specialty training posts led residents to feel a ‘constant pressure’ to gain ‘extra points’ by completing audits, quality improvement projects, teaching and research, whilst moving hospitals frequently. Resident anaesthetists felt that this led to tokenistic projects seen as tick‐box exercises, consuming significant amounts of time and taking them away from valuable clinical training opportunities. The disconnect between the value of performing well in clinical anaesthesia vs. gaining training portfolio points affects work–life balance and satisfaction. Resident anaesthetists stated that they are spending significant amounts of time outside the hospital with non‐clinical commitments including updating their portfolios.

*“The bottleneck … means that your portfolio has to be bigger and better every year, which then means you're spending hours outside of work, just doing your portfolio. I think at one point I worked out that with my shifts and portfolio it was like 100 plus hours a week.”* P042, other resident anaesthetist, female

### Clinical commitments

The clinical role of intensive care medicine was perceived to be a burdensome responsibility for resident anaesthetists who may have only started anaesthesia training recently. Even senior resident anaesthetists described the pressure and responsibility of ICU shifts in negative terms. Questions were raised about the appropriateness of resident anaesthetists providing ICU on‐call cover regularly, particularly for those who are not aiming to pursue a career in intensive care medicine. On‐call shifts were felt to be highly weighted towards service provision with limited learning opportunities. Resident anaesthetists would often cover ICU on‐call shifts when they were not on ICU placements, negatively impacting the number of anaesthesia training lists and clinical cases completed, and sometimes requiring extension of anaesthesia specialty placements.

*“We do a lot of intensive care work, and I don't want to be an intensivist. And all our long days and nights are based in intensive care, and for someone who doesn't enjoy intensive care, I think that definitely affects my view on going into work and my whole anaesthetic training.”* Focus group 1, year 4 resident anaesthetist, male

Obstetric anaesthesia was likewise raised as a clinical stressor due to the high acuity of obstetric care, the proportion of service provision vs. training lists, and the rapidity of reaching a high level of entrustment, which was felt to be disproportionate compared with other curriculum areas.

*“I also think that obstetrics by its nature is quite a scary environment, because patients can get sick quickly, and emergencies can happen very quickly.”* P024, year 4 resident anaesthetist, male

Stress due to rotas and service provision varied with the local environment and rota patterns. Many negative perceptions centred on lack of flexibility to take leave. This caused some resident anaesthetists difficulties in accommodating important life events, even when requested in advance. There were, however, examples of good practice and positive perceptions of anaesthetic rotas, which were felt to be more manageable than other specialities. Some resident anaesthetists described ease in gaining approval for annual leave and the creation of bespoke ‘request rotas’ was beneficial, although the additional work required to implement them was recognised.

*“They have a request rota, that's the first time I've ever worked one; it's wonderful; it definitely creates more work for the people doing the rota, but it's made my life much more manageable”* P021, year 4 resident anaesthetist, male

Frequent night shifts and on‐calls without adequate rest contributed to fatigue, and dissatisfaction with the balance between training time vs. service provision was reported frequently by resident anaesthetists. However, some stakeholders emphasised that an element of service provision is expected within a training contract.

*“I'm supposed to be training but there's quite a lot of bits where there doesn't seem much emphasis on training at all. It's just service provision.”* P073, year 3 resident anaesthetist, male

A lack of autonomy was described by resident anaesthetists, impacting both their personal lives and within the clinical environment. Limited control over placements and rotations was described negatively, with changes sometimes imposed at short notice. Clinically, dissonance was noted between the level of responsibility during out‐of‐hours vs. elective work, whereby resident anaesthetists may independently care for the sickest patients in the hospital at night but are then closely scrutinised during daytime work.

*“Your future is in someone else's hands, it's over to your Training Programme Director organising where you go and things… you suddenly have to move halfway across your deanery, and without warning, away from your children or your partner, or your life … we can feel like a number in a system and that's certainly not good for wellbeing.”* P003, year 2 resident anaesthetist, female

*“I hate being a trainee, I hate everything that goes with it, I hate the fact that you're treated like a child the entire time”* Focus group 1, year 5 resident anaesthetist, female

Formalised entrustment levels were identified as a helpful tool to determine progression to higher levels of independence. This approach led to increased confidence and job satisfaction. Transitioning to less than full‐time training resulted in more control over working hours and shift patterns.

*“Having control over their career and their progress, I think it's amazing that they can all go less than full‐time if they want.”* P056, stakeholder, male

The COVID‐19 pandemic changed the clinical work of resident anaesthetists significantly, in addition to wider social effects. There were unforeseen cancellations of elective work which diminished training opportunities, plus widespread redeployment to support intensive care. Difficult clinical experiences led to some resident anaesthetists suffering significant deterioration in their mental health, including developing post‐traumatic stress disorder. Physical health impacts were described, such as long COVID and back pain related to wearing personal protective equipment for long periods. However, participants noted some significant positive aspects, with improvements to camaraderie within teams, increased focus on wellbeing, and time away from the ‘hamster wheel’ of training.

*“Trainees are really struggling, they've lost a lot of training experience and clinical experience and now trying to catch up on that training and seek out training opportunities in the current climate where a lot of elective lists get cancelled because of the bed issues.”* P051, stakeholder, female

*“During the pandemic I actually would say, completely paradoxically, that it was a huge release for me … stepping off that training bandwagon for a year and a half was the decompression switch that I needed.”* P029, year 4 resident anaesthetist, female

### Structure of training

Recruitment stressors related to difficulties in progressing to specialty training, and the work required to achieve a competitive portfolio for this application. The ‘bottleneck’ between core and speciality training was discussed frequently, with a lack of progression leaving some resident anaesthetists stuck between posts for multiple years, contributing to the decision of some doctors to leave the speciality.

*“Friends I had [in training year 2], lots of them couldn't get jobs third time round, so a lot of them I think quit anaesthetics, some of them became GPs, some of them are still trying.”* P030, year 4 resident anaesthetist, female

There was a lack of understanding as to why anaesthetics is not a run‐through training pathway; it was generally felt that run‐through training would relieve the pressures of specialty recruitment and provide more stability for resident anaesthetists and their families. However, some appreciated the natural break between stage 1 and 2 as an opportunity to pursue alternative experiences.

*“I think I can speak on behalf of almost everyone. It would be nice to have a run‐through where you don't have to stress about getting a ST4 [speciality training] job.”* Focus group 1, year 4 resident anaesthetist, male

*“I really appreciated having a natural break between core training and speciality training because it meant that I could take a couple of years out, without having to ask permission or arrange it with the school or fill out 60 million bits of paper.”* Focus group 1, year 5 resident anaesthetist, female

Some stakeholders held a more positive view towards the specialty recruitment process, describing residents as perceiving the situation to be ‘worse than it is’ based on bad experiences of previous cohorts and late advertisement of available posts. Ongoing improvements were highlighted, such as the expansion of specialty training numbers.

There were significant impacts on resident anaesthetists due to rotational training; large geographical deanery areas; long commutes; and the need to change deaneries for specialty training posts. Some residents felt that they could not ‘set down roots’ or needed to plan their life around rotations. For those undertaking long commutes, this directly impacted their ability to rest, whilst others described the negative effects of staying away from home, ‘living out of a bag’ and leaving family for prolonged periods, leading to isolation from their support systems. Uncertainty over training locations affected resident anaesthetists and their families. Moving between healthcare providers brought recurrent issues with the administrative burden of repeating employment forms.

*“If I want a family, I'm going to have to plan around my rotations, which seems really bizarre.”* P064, year 4 resident anaesthetist, female

*“They made me repeat that paperwork every three months, stuff like that is just nonsense.”* Focus group 1, year 5 resident anaesthetist, female

Resident anaesthetists experienced multiple financial stressors. The nature of rotational training has led to some paying for two separate homes or having the financial burden of long commutes. Although relocation expenses were commended, there were difficulties with expenses being taxed, paid late and insufficient. Some resident anaesthetists described challenges claiming expenses for courses, leading them to pay for these themselves. Concerns regarding the overall cost of training included professional registration fees; examination fees; and revision courses. Pay disputes by doctors, which were ongoing at the time of the study, were raised, with some resident anaesthetists feeling underpaid and underfunded. Some felt disadvantaged by the cost of childcare, noting that it can be more financially viable to work less than pay for a nursery.

*“There was a date… when my relocation expenses came in late… I literally didn't even have £5 to get to the hospital… If I didn't receive the relocation expenses, I would have dropped out of training because I absolutely wouldn't have been able to pay to go to work.”* P010, year 2 resident anaesthetist, male

*“No one goes into this job to be paid well, you know, but people, I think, people like to feel like they're remunerated semi‐fairly”* P065, year 4 resident anaesthetist, female

The transition to the new 2021 RCoA curriculum and ambiguity in the requirements for resident anaesthetists' annual review of competencies have increased stress. During the curriculum change, many resident anaesthetists in the first ‘core’ phase of training described a lack of job security regarding the need for top‐up years to transition to the new curriculum. However, extending core training to three years has allowed residents extra time to sit exams and prepare for speciality recruitment. For stage 2 and 3 residents, there have been issues with transitioning between curricula near completion of training. Generally, moving the curriculum away from the ‘tick‐box’ approach was seen as an improvement, but has led to a lack of clarity, with regional variation in what is now required.

*“There is still a lot of ambiguity about the new curriculum and what it means to get signed off for certain things.”* P023, year 4 resident anaesthetist, male

### Workplace culture

Poor morale was described among both resident anaesthetists and trainers, often due to being overstretched and overworked. Fatigue was a key factor worsening morale and burnout, impacting rates of sickness and interactions between team members. Moreover, a shift of attitude since COVID‐19 has led some consultants to have less enthusiasm for their jobs and for teaching, or to take early retirement, with direct effects on residents' experience of training.

*“I felt that the consultants were really happy, really involved, really wanted to teach, did extra things, yeah. And coming back [after COVID‐19], I felt there's been less of that now because everybody is just so tired.”* P003, year 2 resident anaesthetist, female

Resident anaesthetists often reported feeling underappreciated and undervalued because of workplace culture. With frequent rotations and changing teams, they described feeling like a ‘transient being that holds a bleep’ and a ‘number on a spreadsheet’. Some described a bullying culture within healthcare institutions, with complaints about bullying and harassment not being dealt with appropriately. One participant reported being labelled as a ‘troublesome trainee’ after raising concerns and seeking professional support.

Sex was raised by both female and male participants, who shared the perception that female residents need to work harder to ‘prove themselves’; are treated less seriously; are spoken down to; and given less responsibility than male counterparts. Other resident anaesthetists described issues related to their race or to neurodiversity.

*“It definitely is something that comes across that as women we are treated less professionally, less seriously, and we definitely have to kind of come across a lot more serious to be taken seriously. Even our registrars laugh and say ‘oh you're a girl, you've got to work harder’ and everyone is aware of this.”* P004, year 2 resident anaesthetist, female

Wider health service pressures affecting resident anaesthetists stress included ‘fighting for beds’ in order to start operating lists and being asked to fill rota gaps ‘last minute, all the time’. A changing clinical role was described due to hospitals being busier with sicker patients, and extra work commitments due to the COVID‐19‐related backlog in elective care. Rotational administrative tasks such as employment contracts, registering with payroll, claiming expenses and booking leave were hampered by under‐resourced human resources departments and poor communication with management teams in the NHS.

*“We're dealing with sicker patients, aren't we? And a busy [emergency department], and busier [emergency] lists, and I think out of hours work it's got busier.”* P012, year 3 resident anaesthetist, female

*“I think another thing that could be done a lot better is the lead employer arrangements for trainees.”* P023, year 4 resident anaesthetist, male

We identified supportive factors which included some mechanisms currently in place, and recommendations for improvement.

### Individual factors

Resident anaesthetists discussed the value of social support networks (including friends, family and peers); those with children particularly appreciated practical help with childcare.

*“My mental health is fairly robust, purely because I have quite good support systems.”* P077, year 5 resident anaesthetist, female

Resident anaesthetists also discussed individual actions such as exercise; mindfulness; meditation; and working less than full‐time to relieve stress. Less than full‐time resident anaesthetists reported having an improved quality of life and more time for themselves, family and exercise. Financially, it was reported that reducing the number of working hours could be more economical than working full‐time and having to pay for additional childcare. Training benefits of less than full‐time working included resident anaesthetists feeling more prepared for clinical work; having time to catch up on non‐clinical requirements such as the logbook; moving less frequently; being able to sustain long commutes; reducing their working intensity; and being able to recuperate after on‐calls.

*“Certainly, I felt since going 80%, I've just enjoyed my job a million times more, like far more than 20% more”* Focus group 1, year 4 resident anaesthetist, male

*“I did not see myself continuing at that level of intensity for another half of a decade. So I applied to go less than full‐time … it was go less than full‐time or consider leaving medicine”* P029, year 4 resident anaesthetist, female

### Local factors

Many participants described the approachable and accommodating nature of their educational supervisors, college tutors and anaesthetic consultants. However, educational supervisor support varied depending on the enthusiasm of the supervisor; the quality of the relationship within trainer–trainee dyads; and generational differences in how supervisors and supervisees viewed training. Some supervisors highlighted the challenge of supporting resident anaesthetist wellbeing whilst meeting their training needs. They also noted the difficulties of providing a pastoral role without formal training in mentoring or counselling.

*“I'm an international medical graduate, I don't have anyone else, I don't have family, I don't have a partner. 90% of my support comes from my department.”* P075, year 3 resident anaesthetist, female

It was recognised that resident anaesthetists can access mental health support and coaching through their family doctor, hospital counselling and deanery professional support units (deanery‐led services aiming to support the progression of postgraduate training). Wellbeing initiatives such as mindfulness services were recognised as being ‘well‐intentioned’, but respondents were concerned that they were often not practical or constituted ‘lip service’ or ‘box‐ticking’. Some resident anaesthetists expressed their reluctance to seek help to avoid being stigmatised as having difficulties. Stakeholders discussed taking a proactive approach to identifying resident anaesthetists who might be struggling and acting pre‐emptively.

The provision of rostered teaching time was beneficial, both in terms of the teaching itself and opportunities for networking with peers. Resident anaesthetists valued opportunities for formal and informal sharing of experiences through teaching sessions, mortality and morbidity meetings, buddy schemes and initiatives such as ‘Coffee and a Gas’ [12]. The importance of social spaces was raised, although some resident anaesthetists described having to battle to retain rest facilities.

*"We have a really good social space… it's a fantastic place for consultants to chat with trainees… I think you learn a lot of the time, you realise the problems you're facing aren't just you."* P061, year 2 resident anaesthetist, male

Formal departmental processes described positively by resident anaesthetists included regular forums with college tutors to give feedback and raise concerns, and having wellbeing representatives and a fatigue committee to embed initiatives such as incorporating fatigue checks into handovers. Departments that were accommodating with annual leave, study leave and sick leave requests were appreciated.

### Regional factors

Regional stakeholders discussed the benefits of sharing information and resources for wellbeing across their deanery and regional decisions were noted to impact local practice. Regional teaching programmes were positively received. However, tension was noted between providing online teaching accessible across a wide geographical area and hosting in‐person study days, which provide peer networking opportunities.

*“Our deanery is really good at telling you where you're going to go in advance.”* P065, year 4 resident anaesthetist, female

Professional support units were identified repeatedly as beneficial for coaching, examination help and neurodiversity support. Other regional support schemes including return‐to‐work programmes were well received, but with recommendations to ensure that meetings were planned for before, during and after time away. Another suggestion was to ensure appropriate funding be available for resident anaesthetists to attend return‐to‐work courses and attend operating theatres for a supernumerary period.

*“The person I went to see at the PSU [professional support unit] is the only reason I was in training as long.”* P042, other resident anaesthetist, female

### National factors

The positive impact of national policy and guidance on local practice was noted. Resident anaesthetists described varying levels of awareness of online resources for wellbeing. One recommended a support line, available via professional association and union websites.

*“Removing the stressors is the main thing for us to do as a professional organisation, employers, training body… as opposed to ensuring [trainees] go on a mindfulness course.”* P056, stakeholder, female

Less than full‐time training is a national initiative which has made a significant impact on wellbeing as noted by resident anaesthetists and stakeholders. Increased flexibility in application requirements has led resident anaesthetists to notice a dramatic increase in the number of less than full‐time resident anaesthetists, leading some participants to reflect on what standard full‐time working hours should be.

*“Most of the part‐time colleagues that I work with are much happier than the full‐time ones; I think they just have more time for themselves really.”* P025, year 4 resident anaesthetist, male

### Perspectives on anaesthetic careers

Despite the stressors identified, anaesthetics was generally described as a positive career choice, particularly compared with other medical specialities, due to the support and educational opportunities experienced as a resident anaesthetist and the future work–life balance as a consultant. However, there were still many resident anaesthetists considering leaving the training pathway, the speciality or medicine altogether. Alternative anaesthetic career paths such as speciality and specialist doctor roles (experienced doctors who work in hospital settings within a particular speciality) or taking the portfolio pathway to a consultant post (thereby allowing recognition of expertise acquired without necessarily being in a formal training post) were reported as options offering increased autonomy and flexibility.

*“[Specialty doctors are] not having to move all the time, not having to learn new computer systems all the time, not having to get to know people and by the time you get to know them you just leave again.”* P070, year 3 resident anaesthetist, female

Some resident anaesthetists had considered switching speciality to benefit from more routine hours and fewer rotations. Consideration of alternative careers beyond the NHS was triggered by feedback from colleagues who had already left medicine; the physical demands of working night shifts; harassment at work; and concerns over pay. Comparisons were drawn with training pathways in different countries, specifically regarding the duration of training and degree of autonomy.

*“[In other countries] the contrast is massive, they're doing their own lists, supervising kind of themselves, there's always a consultant that they can ask, there's always a consultant checking in on them, and they seem 1000 times happier with sort of their day‐to‐day anaesthetic practice.”* Focus group 1, year 4 resident anaesthetist, male

## Discussion

We have provided a detailed qualitative analysis of issues related to resident anaesthetists' stress and wellbeing, centring on clinical factors; non‐clinical commitments; the structure of training; and workplace culture. We found that working in ICU and obstetrics were significant clinical stressors and that resident anaesthetists particularly resented covering ICU out‐of‐hours as it negatively affected anaesthesia training opportunities. The main non‐clinical stressors were examinations and the pressure to regularly improve portfolios and update logbooks. Extra time spent on teaching, research, examinations or quality improvement activities led to some resident anaesthetists reporting working over 100 hours per week with their combined clinical and non‐clinical commitments.

Issues related to the structure of training included: the recruitment process to specialty training posts; recent changes in the national anaesthesia curriculum; difficulties with frequent changes to location of training and long commutes; and the financial burden generated from rotational training across large geographical areas. Whilst unfamiliarity with the curriculum was a stressor, the introduction of entrustment scales helped support resident anaesthetists when transitioning to higher levels of responsibility for on‐call anaesthesia duties, by providing a holistic assessment of their capabilities. National training curricula need to develop but our study has highlighted difficulties in transitioning to a new curriculum across the three stages of training in the UK. Participants suggested giving appropriate lead times and notice before making changes to improve this process. Furthermore, a recurrent theme across our study was a dichotomy between the understanding of the issues between resident anaesthetists vs. stakeholders, which highlights that communication should be improved.

Training on a rotational basis can increase travel time and expense, and lead to longer working days, but residents may benefit from diversity of clinical experience, departmental structures and sub‐specialty training in different hospitals. Designing rotations to balance stability with appropriate and timely placements to provide a well‐rounded experience is challenging [7, 13]. We suggest that training programmes should be structured to give resident anaesthetists stability. This could be achieved by reducing rotations between hospitals that can provide similar clinical experiences (e.g. district general hospitals within stage 1 training) whilst giving advanced notice of rotations to specialist centres later in training. Examples of good practice were included in our research, such as Training Programme Directors organising individual meetings with resident anaesthetists at the start of each stage of training to discuss future rotations.

Workplace culture can have a negative impact on resident anaesthetists and trainers, with low morale and risk of burnout worsened by fatigue from busy workloads and on‐call shifts and wider NHS issues. Effects of stress on resident anaesthetists included fatigue; low mood; depression; sleep disturbances; burnout; and reduced clinical performance. Patient safety is an important part of the anaesthetic curriculum and resident anaesthetists are exposed to patient safety incidents, which can have negative effects on both mental and physical health. Robinson et al. interviewed UK resident anaesthetists who had experienced patient safety incidents and stressed the importance of a supportive work environment, structures for debriefing and the need to raise awareness of system‐based approaches to learning from incident investigations [14]. Our study has underlined the need for regular peer‐to‐peer activities, supportive departmental culture, educational supervisor involvement and ‘Coffee and a Gas’ initiatives’ which can provide opportunities to discuss and reflect on challenging clinical cases in a supportive environment.

The support available to resident anaesthetists seems to stem mostly from local and regional sources, bolstered by useful national initiatives instigated by the RCoA, Association of Anaesthetists and NHS Employers, including fatigue guidance and policies on less than full‐time working. Support is embodied in roles such as educational supervisors who were frequently the first port of call for resident anaesthetists, as well as services such as professional support units, mentoring and wellbeing initiatives. A recent systematic review of the international literature on burnout in anaesthesia training also identified protected rest time and restricted working hours as effective strategies to prevent burnout, plus support at individual level through mindfulness, exercise and resilience courses [15].

Previous studies on resident anaesthetists in the UK identified the recruitment process and high non‐clinical workloads as significant stressors [2, 3, 16]. These studies align with the conceptual model presented by Winter et al. in their scoping review, which identified eight external factors impacting anaesthesia residents' wellbeing and stress: training; clinical role; progression; work patterns; resources; rest; support; and cultural factors [1]. The impact of autonomy, the wider culture and the healthcare role have been raised in the conceptual model of clinician wellbeing by Brigham et al. [17], whilst issues with examinations, workplace assessments and challenges securing future positions have been raised in international literature on anaesthetists' wellbeing [18, 19]. A lack of peer‐to‐peer support and socialisation may be a more prevalent issue for anaesthetists than other hospital speciailties. However, fewer concerns appear to have been raised about working conditions and compromised patient care by resident anaesthetists in this study compared with reports from the wider workforce [20].

We have identified supportive factors from an individual to national level, with recommendations suggested for each. This follows the structure of recommendations made by the RCoA following its report on the welfare and morale of resident anaesthetists [3]. Some of these suggestions are already being examined, including via the review of rotational training prompted by the 2023 Extraordinary General Meeting of the RCoA, but there are further improvements to be made. Nationally, the increase in less than full‐time training has implications for workforce planning, and our findings align with wider reports that the reasons for less than full‐time applications are moving more towards supporting wellbeing [21]. We agree with previous recommendations that wellbeing initiatives should focus on external and organisational factors; therefore, government and healthcare bodies have a key responsibility to act [1, 22]. It is vital that these issues be improved as, in addition to negative impacts on individual residents, poor staff wellbeing and stress have been associated with medical errors; increased absenteeism; high staff turnover; and negative outcomes for patient safety and satisfaction [23, 24, 25].

The main strength of this research is that the qualitative study design has produced rich data from a diverse group of participants resulting in a detailed analysis of the issues facing resident anaesthetists. However, we were unable to recruit stakeholder participants from Scotland, Wales and Northern Ireland and so the perspectives of trainers working in the devolved nations are not represented.

There were several contextual factors which are likely to have impacted our study findings. The research was conducted during a time of industrial action due to pay disputes and followed the COVID‐19 pandemic which affected wellbeing across residents and trainers in many specialties, but particularly impacted anaesthesia. Also, it was conducted shortly after a national anaesthetics' curriculum change which affected competition for training places at the specialty level. These external factors brought an added dimension to the research on stressors affecting wellbeing but some of these were transitional in nature.

The research team included three anaesthetists who brought their own lived experiences with them when conducting interviews and focus groups, but the team was balanced with three non‐medical investigators, including our patient partner who was involved at all stages of the study, and ensured we considered the wider implications of the issues identified.

Table 2 outlines our recommendations, based on findings from this research, which seek to improve the experience of resident anaesthetists and support their wellbeing. Due to the variance we found across the UK, there are opportunities to learn from excellent local practices as well as wider national initiatives. Our in‐depth qualitative analyses found that stressors contributing to resident wellbeing related to clinical and non‐clinical workload, structure of training and workplace culture. Whilst there were individual factors affecting wellbeing, most stressors were external, over which residents had little control, and similar to the features identified in the scoping review by Winter et al. of the literature: training; clinical role; progression; work patterns; resources; rest; support; and cultural factors [1].

**Table 2:** Implications for policy and practice.

Level	Recommendation	Benefit
**Individual**	Improve awareness of and engage with support systems	Opportunities to be proactive in self‐care and seek help promptly as needed
**Local**	Provide resources including social facilities, rest spaces, parking and lockers	Spaces for peer to peer and senior support through social facilities. Rest spaces allow for breaks within and following shifts. Provision of parking and lockers can reduce micro‐stressors
	Encourage social initiatives such as ‘Coffee and a Gas’	Opportunities for resident anaesthetists to share their experiences safely and gain informal support
	Rotas should accommodate trainee leave requests; consider the use of request rotas	Resident anaesthetists able to balance life commitments with work more easily to improve wellbeing
	Include wellbeing focus in educational supervisor training	Educational supervisors are aware of local and regional services that resident anaesthetists can be signposted to if needed
	Develop positive departmental cultures	An inclusive and supportive working environment supports resident anaesthetist training and wellbeing
**Regional**	Reduce frequency of rotations and geographical spread	Reduced issues with commuting, stability and the financial burden of training.
	Centralise employment, creating a lead employer for rotations	Reduced issues with payroll mistakes and time taken with administrative tasks when moving Trusts
	Implement regular teaching and training activities aligned to each stage of training	Provide structured learning and an environment for peer interaction, shared learning and reflection
	Increase funding for and raise awareness of professional support units	Provide a vital support service for resident anaesthetists
**National**	Create working group to review ICU on‐call commitments	Maximise resident doctor learning opportunities whilst on anaesthetic placements
	Review recruitment process with consideration of adopting run‐through training	Give assurance and security to resident anaesthetists that there is capacity for their career progression. Increases stability of the anaesthetic resident workforce
	Support and standardise pathways for less than full‐time training	Reduced regional variability with less than full‐time training. Rota pathways should support resident anaesthetists and their departments to ensure adequate staffing levels
	Review examination structure and content	Reduce difficulties that resident anaesthetists have with the quantity and relevance of examination materials

In its report on the welfare and morale of UK doctors in anaesthetic training in 2017, the RCoA underlined the urgent need to ensure that doctors continue their training through a systems‐based approach to safeguard the health and wellbeing of the anaesthetic workforce [3]. Key recommendations emphasised the need to focus on workplace culture; rest facilities; rotas; and flexible working including less than full‐time training. Our study has reiterated the importance of providing a supportive culture at departmental level and identified mechanisms which work well such as shared rest areas; request rotas; ‘Coffee and a Gas’ type initiatives; and peer‐to‐peer training activities. We have also highlighted the significant effect that going less than full‐time has had on the wellbeing of resident anaesthetists when they may be struggling with long commutes and extra stressors through the time pressure of additional non‐clinical commitments. Other changes to the structure of training could make a major difference to the morale and welfare of the resident anaesthetic workforce and we recommend strongly that further research is conducted to understand the benefits, disadvantages and challenges of implementing run through training for anaesthesia; reducing intensive care on call commitments; decreasing frequency and distance of training rotations; and providing a single lead employer for each training rotation.

## Supporting information


**Appendix S1.** Topic guide for resident anaesthetist interviews.

**Appendix S2.** Topic guide for educational stakeholder interviews.

**Appendix S3.** Illustrative quotations for main themes and subthemes.
