# Supplementary material for: A qualitative exploration of stressors in anaesthesia training in the UK and mechanisms to improve resident wellbeing
Source: Anaesthesia. 2025 Feb 25;80(7):799–811. doi: 10.1111/anae.16575 (PMC12171793; doi:10.1111/anae.16575)
Supplement: Supplementary file 1 — Appendix S1. Topic guide for resident anaesthetist interviews. [file ANAE-80-799-s002.docx]

**Appendix S1: Topic guide for resident anaesthetist interviews**

- 1. Introduce self and role, research and funding, university.
  2. Explain confidentiality, recording, expected length of interview, nature of discussion, reporting and data storage/archiving.
  3. Any questions?
  4. Check written consent

Topic guide

1. Can you tell me a little bit about your anaesthetic training to date and current role?
2. What have been the highlights of your training experiences so far?
   1. Why are these the highlights?
3. Have there been any points in your anaesthesia training that you have found particularly stressful or challenging?
   1. Can you describe the challenges? Why was it difficult?
   2. How did you cope with these challenges?
4. Has your mental health been affected by these experiences? If so, how?
5. What are the main pressures anaesthesia trainees face currently?
6. Have you ever felt that you might not continue your training?
   1. When/why?
   2. What happened?
7. How do you feel your wellbeing and mental health has been supported during your training?
   1. Where does support come from? E.g employer/education provider/supervisors/peers?
   2. What do you find most useful in terms of support?
8. Do you think your experiences in training are typical, from discussions with your peers?
   1. Similarities/differences?
9. Have your experiences affected your view on a career in anaesthesia?
10. How much of a priority is wellbeing in anaesthetics training?
11. How can support for trainees’ wellbeing be improved?
12. Is there anything else you would like to add?

- Remind participants that they can get in contact with any queries
- Ask if participants would like to see a copy of the transcript for information
- Remind participants of their right to withdraw
- Signpost to BMA support services
