# Supplementary material for: A qualitative exploration of stressors in anaesthesia training in the UK and mechanisms to improve resident wellbeing
Source: Anaesthesia. 2025 Feb 25;80(7):799–811. doi: 10.1111/anae.16575 (PMC12171793; doi:10.1111/anae.16575)
Supplement: Supplementary file 2 — Appendix S2. Topic guide for educational stakeholder interviews. [file ANAE-80-799-s003.docx]

**Appendix 2: Topic guide for stakeholder interviews**

- 1. Introduce self and role, research and funding, university.
  2. Explain confidentiality, recording, expected length of interview, nature of discussion, reporting and data storage/archiving.
  3. Any questions?
  4. Check written consent

Topic guide

1. Can you tell me a little bit about your professional background and your current role(s)?
2. Are there any points in anaesthesia training that you see as particularly challenging for trainees?
   1. Can you describe the challenges?
3. What are the main stressors that anaesthesia trainees face?
4. Do you think these stressors have changed over time?
5. How do you think these stressors effect trainee’s experiences and perspectives on a career in anaesthesia?
6. How do you feel trainees’ wellbeing and mental health is supported during their training?
   1. Where does support come from? E.g employer/education provider/supervisors/peers?
   2. What do you see as being most useful in terms of support?
7. What barriers are there to supporting trainees’ well-being and mental health?
8. How much of a priority is wellbeing in anaesthetics training?
9. How can support for trainees’ wellbeing be improved? Is there anything else you would like to add?
