# Supplementary material for: A qualitative exploration of stressors in anaesthesia training in the UK and mechanisms to improve resident wellbeing
Source: Anaesthesia. 2025 Feb 25;80(7):799–811. doi: 10.1111/anae.16575 (PMC12171793; doi:10.1111/anae.16575)
Supplement: Supplementary file 3 — Appendix S3. Illustrative quotations for main themes and subthemes. [file ANAE-80-799-s001.docx]

**Appendix S3 - Illustrative quotations for main themes and subthemes**

**Theme*:*** *Non-clinical activities*

| **Subthemes** | **Illustrative quotations**  *(participant identifier, participant role, gender)* |
| --- | --- |
| Revision commitments | “*I think the exam really affected kind of work-life balance, and all of the things that you would normally do to look after your mental health and wellbeing. Like regular exercise, seeing friends, relaxation time, all get taken up by revision*.” (**P069, year 2 resident, female)** |
| Exam relevance | “A*rchaic and not always very relevant to things, not as relevant as they could be*.” **(P029, year 4 resident, female)**  *“It’s an exam where no matter how much reading you do, even if you have read something, it still might not be the right answer.”* **(P021, year 4 resident, male)**  *“The FRCA [Fellowship of the Royal College of Anaesthetists examination] was definitely harder just because of the breadth of knowledge expected and in the level of detail that's required”* **(P021, year 4 resident, male)**  *“It's a lot of esoteric knowledge which doesn't actually help in your day to day*” **(Focus Group 1, year 4 resident, male)** |
| Examination structure | *"You have the viva which is an exam format that you don't really see in undergraduate exams. And so it's having to learn a new way of doing the exam as well. I think that's quite challenging.”* (**P069, year 2 resident, female)**  *“The viva is being cancelled now ... which is definitely a good thing.”*  **(P070, year 3 resident, female)** |
| Examination cost | *“People can drop thousands of pounds trying to pass these exams if they don’t pass it the first time.”*  **(P029, year 4 resident, female)** |
| Impact of examination failure | *“There are going to be some people who fail it two and three times, and that in a very, very success-biased human, causes havoc with their view of themselves.”*  **(Focus Group 2, Training Programme Director, male)**  *“Exams are a particular issue. I have a string of people who've possibly never ever failed anything ... who fail the primary MCQ [Multiple Choice Questionnaire] a couple of times, or a viva, particularly the viva, and then start to view themselves as a total disaster as a human being, spouse, doctor, everything.”* **(Focus Group 2, Training Programme Director, male)** |
| Stakeholder perspective | *“People are revising from question books that are somebody's distorted memory as they walked out of an MCQ many years ago but have been in the question bank and gone round and round but actually they’re nothing to do with the modern exam.”* **(Focus group 2, Head of School, male)**  *“The basis of this is a test of do you understand the devices, equipment, the drugs, the physiology around you, because understanding those prevents some of the awful mistakes I've seen in other parts of medicine that are based around a fundamental misunderstanding of the kit or the drugs people are using.”* **(Focus Group 2, Training Programme Director, male)** |
| LLP/logbook | *“It’s not just necessarily the hours that you work but it’s the time that you spend thinking about work ... trying to keep all the plates spinning with work time, doing your portfolio, your logbook.”* **(P024, year 4 resident, male)**  *“Having to do an audit every year, a QIP [Quality Improvement Project] every year, it’s not easy, especially because you move around so much”* **(P025, year 4 resident, male)**  *“The bottleneck ... means that your portfolio has to be bigger and better every year, which then means you're spending hours outside of work, just doing your portfolio. I think at one point I worked out that with my shifts and portfolio it was like 100 plus hours a week.”* **(P042, other resident, female)** |

**Theme:** *Clinical Activities*

| **Subthemes** | **Illustrative quotations**  *(participant identifier, participant role, gender)* |
| --- | --- |
| Intensive Care Medicine | *“I do not like ICU [ Intensive Care], and I think part of the reason is because at the end of it ... you've done all this work in ICU and then they kind of say to you thanks, cheers, don't want to see you again, and you get on paper absolutely no recognition for it and it impacts a lot on anaesthetic training”* **(Focus group 1, year 4 resident, male)**  *“We do a lot of intensive care work, and I don't want to be an intensivist. And all our long days and nights are based in intensive care, and for someone who doesn't enjoy intensive care, I think that definitely affects my view on going into work and my whole anaesthetic training.”* (**Focus group 1, year 4 resident, male)** |
| Obstetrics | *"I was doing obstetrics as an ST3 [Specialty Trainee] at a tertiary centre, my first set of nights … that was probably the time in my training where I’ve been the most scared."* **(P026, year 4 resident, female)**  *“I also think that obstetrics by its nature is quite a scary environment, because patients can get sick quickly, and emergencies can happen very quickly.”* **(P024, year 4 resident, male)**  *“The entrustment level set for obstetrics is actually disproportionate to the entrustment levels we have for other areas of the curriculum."* **(Focus group 2, Training Programme Director, female)** |
| Rotas | *“There is a current trainee who’d actually given the department 6 months’ notice that she is getting married … and then, on the day of the wedding, she's been told you have to do the night shift.”* (**P052, stakeholder, male)**  *“Every two weeks I have a set of nights, so it takes me about to the next week to get over it and then I’ve only got a few days before I'm back onto nights.”* **(P021, year 4 resident, male)**  *“They have a request rota, that’s the first time I’ve ever worked one, it’s wonderful, it definitely creates more work for the people doing the rota, but it’s made my life much more manageable”* **(P021, year 4 resident, male)** |
| Service provision | *“I'm supposed to be training but there's quite a lot of bits where there doesn't seem much emphasis on training at all. It's just service provision.”* **(P073, year 3 resident, male)**  *“[Trainees] don't want to be intensive care doctors, they may not want to be obstetric doctors, they want to give anaesthetics, but the way that they are paid, and the way that their contract’s written, is that they do have to provide some service”.* **(Focus group 2, Head of School, male)** |
| Autonomy | *“Your future is in someone else's hands, it’s over to your Training Programme Director organising where you go and things... you suddenly have to move halfway across your deanery, and without warning, away from your children or your partner, or your life ... we can feel like a number in a system and that's certainly not good for wellbeing.”*  **(P003, year 2 resident, female)**  *“The frustration is at night we let them loose to look after the sickest patients in the hospital on intensive care, retrieving patients, transferring patients, massive obstetric haemorrhages. And then during the day we like, oh, you've tied that in wrong. So, I think that there's a dissonance there; what we expect at nighttime, and what we do during the day.”* **(P056, stakeholder, male)**  *“You can see that your independence and autonomy increases as you progress through, so there's a real tangible, different supervisory level and entrustment level depending on how confident your supervisors feel in you. That’s very rewarding, for when you actually are managing a lot more cases independently and doing things that you weren’t able to 6 months or 12 months ago, and that's been really good.”* **(P006, year 2 resident, male)**  *“Having control over their career and their progress, I think it's amazing that they can all go less than full time if they want.”* **(P056, stakeholder, male)**  *“I hate being a trainee, I hate everything that goes with it, I hate the fact that you're treated like a child the entire time”* **(Focus group 1, year 5 resident, female)** |
| COVID-19 | *“Trainees are really struggling, they've lost a lot of training experience and clinical experience, and now trying to catch up on that training and seek out training opportunities in the current climate where a lot of elective lists get cancelled because of the bed issues.”* **(P051, stakeholder, female**)  *“I think there was a time during and just after COVID, when lots of departments were doing loads of wellbeing things and wellbeing was this big thing.”* **(P023, year 4 resident, male)**  *“I think it brought everyone together as a department, and you had a big appreciation for each other more than you might have before.”* **(P022, year 4 resident, male)**  *“During the pandemic I actually would say, completely paradoxically, that it was a huge release for me ... stepping off that training bandwagon for a year and a half was the decompression switch that I needed.”* **(P029, year 4 resident, female)** |

**Theme:** *Structure of training*

| **Subthemes** | **Illustrative quotations**  *(participant identifier, participant role, gender)* |
| --- | --- |
| Changes to curriculum | *“There is still a lot of ambiguity about the new curriculum and what it means to get signed off for certain things.”* **(P023, year 4 resident, male)**  *“People that are approaching the Certification of Completion of Training (CCT) and then have had to go through 2 different curriculums at the end point in their career.”* **(P006, year 2 resident, male)**  *“The year above me feel, I know, felt really let down in that they went through being told there won't be a job for you and a lot of them were almost told ‘sort yourself out for a year’.”* **(P073, year 3 resident, male)** |
| Location and commuting | *“I don't think I've woken up later than half past 5 ever in the last 3 years... I found quite draining. I feel by the time the weekend comes all I want to do is sleep."* **(P005, year 2 resident, female)**  *"If I want a family, I'm going to have to plan around my rotations, which seems really bizarre."* **(P064, year 4 resident, female)**  *"It's a bit rubbish leaving my partner and dog at home on the Monday potentially knowing that you might be staying down there till Friday."* **(P006, year 2 resident, male)**  *“They made me repeat that paperwork every three months, stuff like that is just nonsense.”* **(Focus group 1, year 5 resident, female)** |
| Financial | *"There was a date... when my relocation expenses came in late... I literally didn't even have £5 to get to the hospital [...] If I didn't receive the relocation expenses, I would have dropped out of training because I absolutely wouldn’t have been able to pay to go to work."* **(P010, year 2 resident, male)**  *"No one goes into this job to be paid well, you know, but people, I think, people like to feel like they're remunerated semi-fairly"* **(P065, year 4 resident, female)**  *"I think the main thing that can make a difference is money."* **(P065, year 4 resident, female)**  *“We’re massively disadvantaged in terms of childcare costs and stuff like that...it’s cheaper to take the time off and look after the kids."* **(P021, year 4 resident, male)** |
| Recruitment | *“The biggest problem in training for me, it’s simply that you can’t set down roots”* **(P010, year 2 resident, male)**  *“I'm not denying there isn't a bottleneck, but I know how many jobs I’m releasing, and I know there's expansion coming, and we're communicating it. But I still think [trainees] perceive the situation to be worse than it is.”* **(P050, stakeholder, female)**  *“The biggest other single issue is splitting the training into core and specialty.”* **(P021, year 4 resident, male)**  *“Loads of my colleagues are all now just stuck at staff grade level because they can't get ST4 [Specialty Trainee] jobs.”* **(P064, year 4 resident, female)**  *“There's definitely a feeling amongst everyone that registrar training places are very limited, and the competition is intense, [...] I think that's a stressful feeling, knowing that it's a bit of an artificial situation.”* **(P061, year 2 resident, male)**  *“Friends I had in CT2 [Core Trainee], lots of them couldn’t get jobs third time round, so a lot of them I think quit anaesthetics, some of them became GPs, some of them are still trying."* **(P030, year 4 resident, female)**  *“I think I can speak on behalf of almost everyone. It would be nice to have a run through where you don't have to stress about getting a ST4 job.”* **(Focus group 1, year 4 resident, male)**  *“I really appreciated having a natural break between CT2 [Core Trainee] and ST3 [specialty trainee] because it meant that I could take a couple of years out, without having to ask permission or arrange it with the school, or fill out 60 million bits of paper.”* **(Focus group 1, year 5 resident, female)** |

**Theme:** *Workplace culture*

| **Subthemes** | **Illustrative quotations**  *(participant identifier, participant role, gender)* |
| --- | --- |
| Workplace culture | *“A bit of healthy debate is good but there are some consultants who they’ll ask you why you did this, do you do that, just to kind of introduce a debate about anaesthetic practice, but sometimes they can cross the line, which can be quite harmful.”* **(P008, year 3 resident, male)**  *“It definitely is something that comes across that as women we are treated less professionally, less seriously, and we definitely have to kind of come across a lot more serious to be taken seriously. Even our registrars laugh and say ‘oh you’re a girl, you’ve got to work harder’ and everyone is aware of this.”* **(P004, year 2 resident, female)**  *“I think if you show weakness in the NHS [National Health Service], you stand a very good chance of being bullied.”* **(P029, year 4 resident, female)** |
| Morale and burnout | *“Work just feels relentless now and I don't remember it being like this before.”* **(P033, year 5 resident, male)**  *“I felt that the consultants were really happy, really involved, really wanted to teach, did extra things, yeah. And coming back [after Covid], I felt there's been less of that now because everybody is just so tired.”* **(P003, year 2 resident, female)**  *"It's the people that you are working with are fed up and a bit bummed with the system. Then they're not very positive about that work, or you know, or teaching you.”* **(P065, year 4 resident, female)**  *“Consultants are not as happy as they used to be, and that is kind of filtering down a little bit that their enthusiasm isn’t as high.”* **(P003, Year 2 Resident, female)** |
| Wider NHS issues | *“I think another thing that could be done a lot better is the lead employer arrangements for trainees.”* **(P023, year 4 resident, male)**  *“I think that a lot of the time my main gripes are with the structure of the management systems of the NHS.”* **(P023, year 4 resident, female)**  *“When you're fighting for beds, you know you don't know if your list is going to start.”* **(P040, year 5 resident, female)**  *“We have the waiting list backlog. And the consultants are doing a lot more lists, longer days, more days in a row. But that comes with fatigue and sickness.”* **(P003, year 2 resident, female)**  *“We're dealing with sicker patients, aren't we? And a busy ED [Emergency Department], and busier CEPOD [Confidential Enquiry into Patient Outcome and Deaths] lists, and I think out of hours work it's got busier.”* **(P012, year 3 resident, female)** |

**Theme:** *Effects of stress*

| Subthemes | Illustrative quotations  *(participant identifier, participant role, gender)* |
| --- | --- |
| Effects of stress on trainees | *“I was constantly exhausted. You can’t think straight* *a lot of the time, talk or enjoy doing the work you’re doing. You hate going to work, feeling low in mood and kind of stressed, and like small things just stress you out and I get irritable very quickly."* **(P030, year 4 resident, female)**  *“I would say borderline depression, there’s definitely points where my partner has said you’re definitely not getting far off [depression], you’ve stopped sleeping well, stopped getting joy out of things, they’re major flags.”* (**P021, year 4 resident, male)** |
| Effects of stress on trainees’ family | *“I've never been away from my children as much as I am now. They still cry when I leave, and it's really distressing.”* **(P010, year 2 resident, male)**  *“I was feeling exhausted, not seeing my fiancé, not seeing family, and missing so many family events for various bits and pieces.”* **(P042, other resident, female)** |
| Effects of stress on clinical work | *“I guess more fatigue, but generally exhaustion, some cases I see at work, I don’t care, I just want to get the next patient in and get going with the list rather than worrying about whether someone has filled in a certain bit of paperwork.”* **(P009, year 2 resident, male)** |

**Theme:** *Supportive factors*

| **Subthemes** | **Illustrative quotations**  *(participant identifier, participant role, gender)* |
| --- | --- |
| Individual | *"My partner is very supportive so that makes life very easy."* **(P073, year 3 resident, male)**  *“My mental health is fairly robust, purely because I have quite good support systems.”*  **(P077, year 5 resident, female)** |
| Local: General | *"I'm an international medical graduate, I don't have anyone else, I don’t have family, I don't have a partner. 90% of my support comes from my department."* **(P075, year 3 resident, female)** |
| Local: Educational supervisors | *“You get randomly allocated your supervisor at the beginning of a year, and who knows if you're going to get on, and they're a stranger to you. I always wonder if you really wanted to open up to your supervisor, how many people feel that they can and want to, it's a bit of a strange dynamic.”* **(P003, year 2 resident, female)**  *"My educational supervisor is amazingly supportive”* **(P012, year 3 resident, female)**  *"We aren't specialised*... *we haven't done mentoring qualifications/counselling."* **(P051, stakeholder, female)** |
| Local: Social spaces | *"We have a really good social space.. it's a fantastic place for consultants to chat with trainees.. I think you learn a lot of the time, you realise the problems you're facing aren't just you."* **(P061, year 2 resident, male)**  *"You can gauge other people are having the same worries and experiences."* (**P069, year 2 resident, female)** |
| Regional | *"Our deanery are really good at telling you where you're going to go in advance."* **(P065, year 4 resident, female)**  *"I got PSU [Professional Support Unit] support about dyslexia."* **(P039, year 5 resident, female)**  *"I had some coaching from an exam coach, which was really really helpful."* **(P026, year 4 resident, female)**  *"The person I went to see at the PSU as the only reason I was in training as long."* **(P042, other resident, female)** |
| National: General | *"With the college/schools its a balance.. needing us to achieve all these things to get that certification and balancing the effect that those have on people’s mental health."* **(P012, year 3 resident, female)**  *"The association has quite a lot of resources and counselling services."* **(P050, stakeholder, female)**  *"Removing the stresses is the main thing for us to do as a professional organization, employers, training body ... as opposed to ensuring [trainees] go on a mindfulness course or a tai chi course."* **(P056, stakeholder, male)** |
| National: Less than full time training | *“Certainly I felt since going 80%, I've just enjoyed my job a million times more, like far more than 20% more."* **(Focus 1 group, year 4 resident, male)**  *" most of the part time colleagues that I work with are much happier than the full time ones, I think they just have more time for themselves really”* **(P025, year 4 resident, male)**  *“I did not see myself continuing at that level of intensity for another half of a decade. So I applied to go less than full time ... it was go less than full time or consider leaving medicine”* **(P029, year 4 resident, female)**  *"I'm finding I have no time for exercise at all...the primary reason I want to go LTFT [less than full time] is to be able to go on my bike... it's really important for my wellbeing to do exercise"* **(P045, other resident, male)** |

***Theme:*** *Perspectives on anaesthetic careers*

| **Subthemes** | **Illustrative quotes**  *(participant identifier, participant role, gender)* |
| --- | --- |
| Perspectives on anaesthetic careers | *"Everyone that is a consultant generally paints quite a pretty picture in terms of their stress levels and their work-life balance.”* **(P006, year 2 resident, male)**  *“I think we're very fortunate as anaesthetists."* **(P022, year 4 resident, male)**  *"Anaesthetics as a career for me is the best job in medicine."* **(P010, year 2 resident, male)**  *"There's definitely a team spirit within anaesthetics that I think is probably lacking in other specialties."* **(P061, year 2 resident, male)** |
| Alternative anaesthetic careers | *“[Abroad] the contrast is massive, they're doing their own lists, supervising kind of themselves, there's always a consultant that they can ask, there’s always a consultant checking in on them, and they seem 1000 times happier with sort of their day-to-day anaesthetic practice.”* **(Focus group 1, year 4 resident, male)**  *“[The benefits of specialty doctor careers are] not having to move all the time, not having to learn new computer systems all the time, not having to get to know people and by the time you get to know them you just leave again.”* **(P070, year 3 resident, female)** |
| Alternative careers outside of anaesthetics | *“My wife is a management consultant, and they take medics on at senior associate level, which is good pay [..] and the way that company treats her, it's just like a different world compared to the NHS. I was going to leave, do that and see if I missed medicine.”* **(P045, other resident, male)**  *“I'm definitely looking into potentially consulting in the pharmaceutical industry."* **(P074, year 3 resident, male)**  *“I have looked into applying for something where it is a 9-5 job, like pathology.”* **(P074, year 3 resident, male)** |
